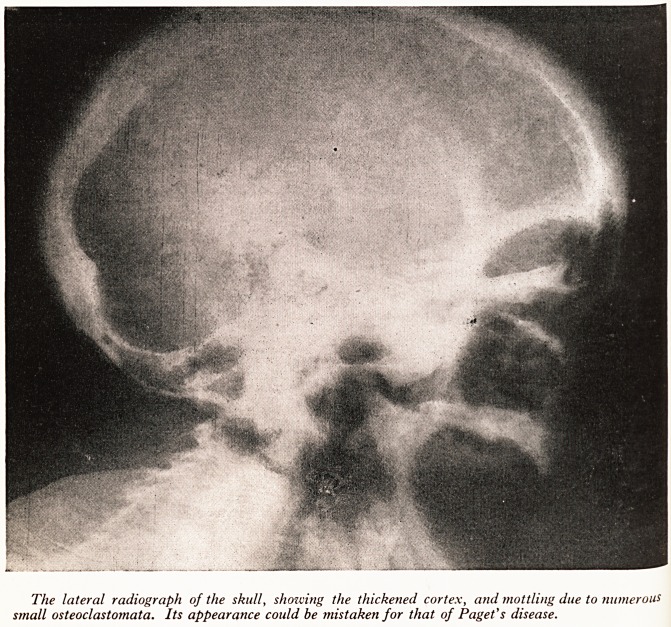# Hypercalcaemia with Hypocalcuria, an Unusual Case of Primary Hyperparathyroidism

**Published:** 1956-07

**Authors:** H. A. W. Forbes, M. J. Wodzinski

**Affiliations:** Physician, S. Devon & E. Cornwall Hospital; Asst. Pathologist, Walton Hospital, Liverpool


### HYPERCALCAEMIA WITH HYPOCALCURIA
### AN UNUSUAL CASE OF PRIMARY HYPERPARATHYROIDISM

BY

H. A. W. FORBES

*Physician, S. Devon & E. Cornwall Hospital*

AND

M. J. WODZINSKI,

*Asst. Pathologist, Walton Hospital, Liverpool*

The importance of this case lies in its great rarity, and the presence of hypercalcaemia with a low urinary output of calcium in hyperparathyroidism due to parathyroid adenoma which was proven by autopsy. Only three other similar cases have been described.

### THE CASE-HISTORY

Miss Ellen H., aged 74, had had rheumatoid arthritis which began in her thirties, but had not been troublesome for many years. Aged 39 she knocked her right breast in which a tender lump developed, and the whole breast grew larger than the other. For this condition she had a radical mastectomy at the age of 45.

She was in good health until, four years before admission, spinal curvature gradually developed, and she noticed weakness first in the right leg and later in both. The weakness and bowing of the spine increased steadily. One year before admission intermittent aching pains in the back and legs began and grew worse. Six months later she became bedridden because of the weakness of her legs. Control of the sphincters remained normal. She lost flesh and the spinal deformity and pains increased in severity.

Mentally unclouded, and suffering her disabilities with fortitude, she was, like her siblings, only about five feet tall, and had a severe kyphoscoliosis. Her ribs were collapsed inwards narrowing the chest from side to side, and the sternum and clavicles were arched forwards. Her head seemed disproportionately large, but was 21½ inches in circumference. The thyroid gland was not enlarged, and no other abnormal mass was palpable in the neck. There was a generalized wasting of muscle and fat. Her legs were paralysed with increased tendon reflexes, extensor plantar responses and sustained ankle clonus. A bony lump was palpable on the left shin. Her upper limbs were weak and spastic. Her hands showed evidence of burnt-out rheumatoid arthritis, with enlarged joints and ulnar deviation of the fingers. Both radii were curved with the concavity forwards. No abnormality was found in the abdomen, respiratory and cardiovascular systems. The arteries were supple and the blood pressure 145/90 mm. Hg. At no time was albumen found in the urine whose specific gravity ranged from 1006–1020.

The following investigations were done:

| | |
|---|---|
| Serum Calcium | 14·9 mg. per 100 c.c. |
| Serum Inorganic Phosphorus | 2·7 mg. per 100 c.c. |
| Serum Alkaline Phosphatase (K.A. Units) | 60 units per 100 c.c. |
| Serum Albumen | 3·7 g. per 100 c.c. |
| Serum Globulin | 2·1 g. |
| Thymol Turbidity Test | 2·7 units. |
| Colloidal Gold Test | 1 + |

| | |
|---|---|
| Blood Urea | 58 mg. per 100 c.c. |
| W.R. and Kahn Reactions | Negative. |
| Serum Calcium | 14·3 m.g. |
| Serum Ionic Calcium | 7·5 mg. |
| Urea Concentration Test | 12% of normal. |
| Plasma Chlorides | 573 mg. per 100 c.c. |
| Plasma $CO_2$ Capacity | 64 vols. |

On three occasions in one week Barney and Sulkowitch's Test gave the faintest cloud indicating Calcium excretion within normal limits.

Radiographs of the bones showed generalized decalcification. A few areas appeared normal, but the whole skeleton was affected, either by abnormal curving, or by scattered rarefied areas due to osteoclastomata, and by thickening or thinning of the bony cortex. Most of the large bones showed all these features. (Plate VIII (*a*) and (*b*).)

The urinary calcium excretion was now studied (see Table I). The patient was given a low-calcium diet which suited her palate. She kept rigidly to it from 10th August to 7th September. A day's supply of food was ashed, and contained 74 mg. of calcium and 234 mg. of phosphorus. She drank exactly 30 oz. of fluid a day until 27th August 1951 when she was urged to drink as much as she could to see if more calcium could be excreted. As the water in Birmingham is very soft calcium intake from this source was negligible.

TABLE I

| Date | Fluid Intake in oz. | Fluid Output in c.c. | 24 hr. Urinary Excretion | | Serum | | |
|---|---|---|---|---|---|---|---|
| | | | Calcium mg./day | Phosphorus mg./day | Calcium mg.% | Phosphorus mg.% | Alkaline Phosphatase K.A. Units per 100 ml. |
| Aug. | | | | | | | |
| 9th | — | — | — | — | 14·2 | 3 | — |
| 16th | 30 | 840 | 40 | 215 | — | — | — |
| 17th | 30 | 890 | 55 | 332 | 15·3 | 2·6 | 52 |
| 24th | 30 | 800 | 41 | — | — | — | — |
| 27th | 41 | — | — | — | — | — | — |
| 28th | 39 | — | — | — | — | — | — |
| 29th | 41 | — | — | — | — | — | — |
| 30th | 87 | — | — | — | — | — | — |
| 31st | 67 | — | — | — | — | — | — |
| Sept. | | | | | | | |
| 1st | 41 | 1260 | 61 | — | — | — | — |
| 2nd | 53 | — | — | — | — | — | — |
| 3rd | 49 | — | — | — | — | — | — |
| 4th | 82 | 1100 | 64 | — | — | — | — |
| 5th | — | — | — | — | — | — | — |
| 6th | 51 | — | — | — | — | — | — |
| 7th | 47 | — | — | — | 14·9 | 2·2 | 55 |

There was a steady increase of her disabilities. First one then the other femoral vein thrombosed, and she grew weaker and more bent. She died quietly and unexpectedly.

### THE AUTOPSY FINDINGS

The examination, 53 hours after death, revealed a marked porosis of the bones which cut with a saw like rotten wood. Both femora showed moderate thickening of the cortex and dilation of the shafts in the upper thirds by copious red marrow tissue. The calvarium was thickened and could be cut by a knife with moderate pressure. The brain, apart from two areas of old symmetrical softening in the basal ganglia, and

PLATE VIII (*a*)

*This radiograph of the right humerus, chest, and spine illustrates the bowing of the bones (spine clavicle and scapula), the variation in thickness of the cortex, and the osteoclastomata in the head and shaft of the humerus and tip of the scapula.*

*The lateral radiograph of the skull, showing the thickened cortex, and mottling due to numerous small osteoclastomata. Its appearance could be mistaken for that of Paget's disease.*

moderate atheroma of the cerebral arteries, was of normal appearance. The thyroid gland had a small adenoma in the upper pole of the right lobe, but was otherwise normal. On the lower lateral aspect of its right lobe was a soft capsulated tumour measuring $3 \times 2\frac{1}{2} \times 2\frac{1}{2}$ cm. The cut surface of this tumour was a deep brownish red colour. No other parathyroid glands were found. The lungs were emphysematous and showed a confluent bronchopneumonia in the right lower lobe. There was atheroma of the coronary arteries and aorta but the myocardium, apart from a few minute scars in the left ventricle was normal.

The liver, spleen, pancreas and suprarenals were normal. Both kidneys were reduced in size and weighed 142 g. The surface was granular. The cortices were narrow and not well differentiated from the medullae. There were several minute, finely granular patches of metastatic calcification on the cut surfaces. The rest of the urinary tract and the other organs of the body were normal.

Sections showed the parathyroid adenoma to have an acinar structure supported by a network of capillaries. The cells were polygonal with finely vacuolated and slightly acidophil cytoplasm and round deeply stained nuclei. Other parts did not have any particular arrangement and the cells were round with watery, slightly basophil cytoplasm, and distinct round or slightly oval nuclei. There were also a few minute islets of small oxyphil cells with pinkish cytoplasm. Kariokinetic figures were found in one or two cells only. Very occasionally some of the ballooned principal cells had two nuclei.

Sections of the kidneys showed thickening of the arteries, and fibrosis of some glomeruli with slight surrounding infiltration by small lymphocytes. There were scattered areas of calcium deposits mainly confined to the medulla. Sections of the stomach, lungs, suprarenals, pancreas, and heart did not show any metastatic calcification.

In the femur there was great osteoclastic activity. The cortex was reduced to slender trabeculae embedded in fibrous marrow, which contained nodules of osteoclastomata. There was no evidence of delay in calcification such as occurs in vitamin D deficiency.

There was no evidence of an attempt to restore new bone. In the sections of bone examined there was nothing to suggest the presence of Paget's disease.

### DISCUSSION

In primary hyperparathyroidism there is usually one adenoma, rarely more, and occasionally hyperplasia of all four glands. The excretion of parathormone by this neoplasia (whether fulminating, steadily progressive, or fluctuating), the calcium intake, and the presence of other diseases, determine the mode of presentation and the course of the disorder.

If the adenoma produces rapidly increasing amounts of parathyroid hormone the patient will die suddenly with great muscular weakness, and anuria (Dawson and Struthers, 1923), but this is exceedingly rare.

Usually the disease runs a fairly steady course, and if it presents before the renal lesions have become severe, the way in which it does so probably depends upon the calcium intake of the patient, who, if he likes milk will develop urinary stones (Albright and Bauer, 1934). If he has a low calcium intake the bony softening will predominate, with pain, deformity, fractures, and ultimately paralysis from spinal cord compression. In all cases calcium phosphate is deposited in the tubules and its presence leads to fibrosis with destruction of the kidney. Subjects with severe renal calcinosis may present with uraemia, and an example of this is reported by Downs and Scott, 1941. In life this patient's serum calcium was low and phosphorus high, and he had a low urinary excretion of calcium. At autopsy an adenoma of one, and hyperplasia of the remaining three parathyroid glands was found. The kidneys had gross calcinosis and fibrosis, but the bones were little affected. It is considered that the adenoma was the initial lesion, and caused the calcinosis, which eventually produced renal failure which in turn induced the other parathyoid glands to hypertrophy. (Ham 1940, Snapper 1949.)

It is possible that some of the few cases reported of patients with proven parathyroid adenomata and bony decalcification in whom the urinary calcium excretion is low may be progressing to the same end as the case mentioned above. A low or normal urinary calcium excretion in the presence of hypercalcaemia may seem incongruous, but it has previously been reported in five cases. Two of these had parathyroid adenomata (Snapper 1949, and Gutman and Parsons 1938, Case 2), and one hyperplasia of all glands in which the main cell was of a type found usually in secondary hyperplasia, though the authors doubt that the kidney lesion could have been primary (Pratt, Green and Neuhauser 1947). The other two cases were sisters with renal rickets, one of which was shown to have renal calcinosis and fibrosis at autopsy (Chown 1936). Two cases of Albright's *et al.* (1934) with proven parathyroid adenomata and poor renal function tests had urinary excretions of calcium which though raised were much lower than usual in hyperparathyroidism. These cases had a greater faecal calcium excretion than normal. The authors suggest that this may be compensatory in cases where renal failure reduces calcium excretion in the urine. The phenomenon of hypocalciuria with hypercalcaemia, is rare, and the mechanism unknown, but a characteristic of the three cases who came to post-mortem examination, or who had renal function tests done, was severe kidney failure, and this might provide a simple *a priori* explanation. It should be pointed out however that many cases of primary hyperparathyroidism with comparably bad renal function tests do excrete excessive amounts of calcium. Snapper 1949 explains the phenomenon by postulating a vitamin D deficiency, common in northern China where he observed his case. Since in the osteomalacia of this region a very low urinary calcium has been observed when the blood calcium was normal (Liu *et al.*, 1940), he suggests that in hypovitaminosis D and hypercalcaemia from hyperparathyroidism, low excretion of calcium in the urine might occur. Adequate renal function tests were not reported in this case history. This type of osteomalacia has not been seen outside northern China (Albright and Reifenstein, 1948). It is therefore not a likely explanation.

The disease may run a fluctuating course (Willich, 1920, and Linden, 1934) and this provides an explanation of some of the cases in which one bone may have the appearance of Paget's disease on X-ray (Albright, 1934, and Gutman and Parsons, 1938) while the remainder of the skeleton is typical of hyperparathyroidism. (See Plate VIII (*b*).) Recalcification in periods of normality could produce the appearances described. Gutman and Parsons (1938) do, however, report a case in whom the two diseases were probably present together, for the alkaline phosphatase remained high for two years after the adenoma had been removed, which is unusual. Albright and Reifenstein (1948) have seen others.

The case presented is of proven primary parathyroid adenoma with hypercalcaemia and a consistently low urinary calcium excretion. In life her kidney function tests showed poor function and at autopsy there was severe renal fibrosis from calcinosis. She fits with the other cases quoted above into the natural history of hyperparathyroidism illustrated in Table II. The present case and those culled from the literature show that though a high urinary excretion of calcium is induced by excessive production of parathormone, this may not always occur in cases of hyperparathyroidism with severe renal defect, and should not be used as a rigid diagnostic criterion when the latter is present.

### SUMMARY

The natural history of primary hyperparathyroidism is discussed, and a case of primary parathyroid adenoma with hypercalcaemia but low urinary calcium excretion is described.

Our thanks are due to Mr. R. P. S. Kelman and Dr. G. G. Gillam of Selly Oak Hospital, Birmingham for permission to publish this case.

TABLE II

### THE NATURAL HISTORY OF PRIMARY HYPERPARATHYROIDISM

### *BIBLIOGRAPHY*

Albright. F., and Bauer, W. (1934). *J. Amer. Med. Ass.*, **102**, 1276.
Albright, F., *et al.* (1934). *Amer. J. Med. Sci.*, **187**, 49.
Albright, F., and Reifenstein, E. C. (1948). *The Parathyroid Glands and Metabolic Bone Disease.* (Baltimore.)
Chown, D. (1936). *Brit. J. Surg.*, **23**, 552.
Dawson, J. W., and Struthers, J. W. (1923). *Edin, Med. J.*, **30**, 421.
Downs, R. S., and Scott, S. (1941). *Arch. Int. Med.*, **67**, 658.
Gutman, A. B., and Parsons, W. B. (1938). *Ann. Int. Med.*, **12**, 13.
Ham, A. W. (1940). *Amer. J. Path.*, **16**, 277.
Linden, O. (1934). *Acta radiol.*, **15**, 202.
Liu, S. H., *et al.* (1940). *Chinese Med. J.*, **58**, 141.
Pratt, E. L., *et al.* (1947). *J. Paed.*, **30**, 388.
Snapper, I. (1949) *Med. Clinics on Bone Disease. pp.* 46 and 91 (*New York*).
Willich, T. (1929). *Bettr. z. Klin. Chir.*, **146**, 103.

## Figures and Tables

**Figure f1:**
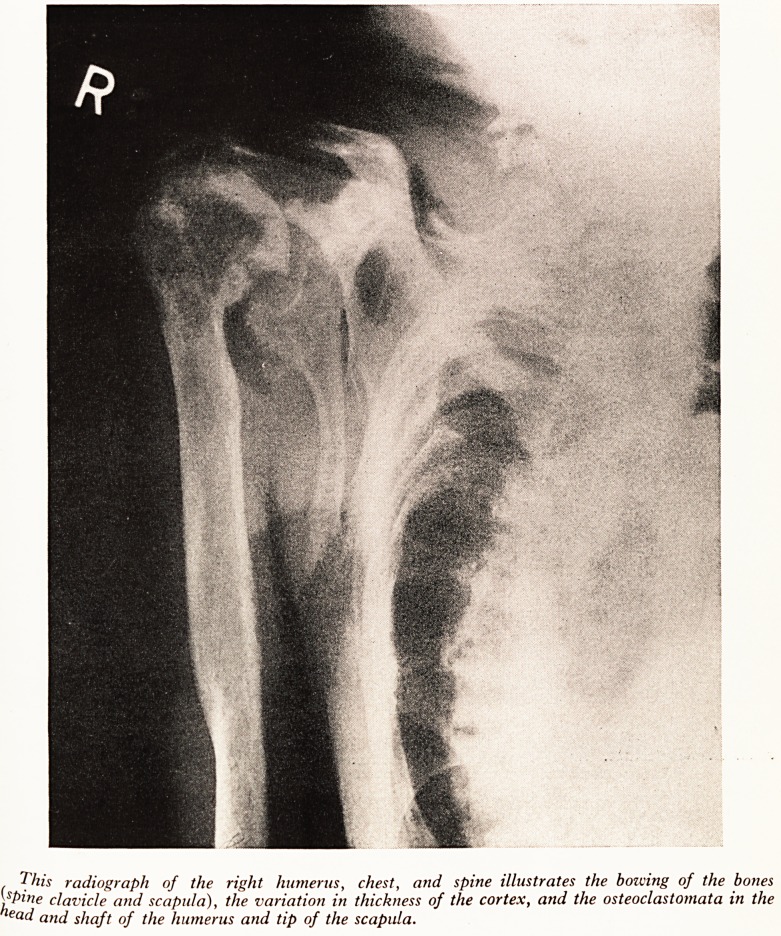


**Figure f2:**